# Aconite

**Published:** 1877-06-20

**Authors:** I. J. M. Goss

**Affiliations:** Marietta, Ga., Author of late work on Materia Medica, etc.


### ACONITE.

By I. J. M. GOSS, A M., M. D., Marietta, Ga.,
Author of late work on Materia Medica, etc.

The medical effects of aconite are just beginning to be appreciated. Says Dr. Sydney Ruiger: "The virtues of aconite are far from being appreciated, but I predict that, ere long, it will be extensively employed. Perhaps no drug is more valuable. Given in large doses, aconite at first produces a sensation of warmth at the pit of the stomach, not unfrequently with nausea and vomiting. This sensation of warmth soon spreads over the body, and it then produces a tingling of the lips, tongue, and adjacent parts; the uvula and tongue feel as if swollen, and deglutition is frequent. A still larger dose induces a numbness, and tingling at the ends of the fingers, rapidly spreading over the entire body, producing more or less anæsthesia and muscular debility, and, if the dose be very large, this muscular debility will be extreme. But in doses of one or two drops of the tincture (saturated), its action on the circulation is very prompt. The above dose, repeated every one or two hours, will frequently reduce the pulse down from 120 or 100 to 60 or 70 per minute, but larger doses, repeated too often, produce such debility of the heart that it causes it to beat much faster, and sometimes it becomes irregular. Whether the pulse becomes faster or slower under the influence of this drug, it always looses strength, which shows the power of it over the heart's action.

Dr. Achscharumow and Dr. Fothergill, in their experiments upon frogs, show that aconite will produce paralysis of the heart, arresting the contractions of the diastole. It affects the respiratory movement in a similar manner; moderate doses render the respiration slower, but large and toxical doses often produce short and hurried breathing. It first stimulates the inhibitory centre of the pneumogastric nerve, and thus slows the action of the heart, and, if increased in dose largely, it finally exhausts the pneumogastric, and, at last, paralyses it, and then the heart beats quicker and irregularly. Its power of controlling inflammation is almost marvelous. It will not always remove the products of inflammation, but, by controlling the inflammatory process, it will prevent the further formation, thus saving the tissues from any further injury. It should always be resorted to in the early stages of inflammation, at which time it is most serviceable. In pharyngitis, tonsilitis, and glossitis, it is an indispensable remedy, and may be given, and locally applied, in the form of a dilution. Aconite not only lessens the frequency of the circulation, thereby decreasing the chemical changes, diminishing the oxidation of the blood, but it increases the flow of blood to the skin, producing perspiration, and, thereby, the rapid radiation of heat, and the evaporization from the body, which also lessens the heat. And not only does aconite diminish the heat thus, but also by its direct influence on the vascular system. It is well known that the vascular system is always in a state of semi-contraction in fevers and inflammation. Aconite dilates the arterioles, and increases the capacity of the vascular system, and thereby drains the blood away from the inflamed organ. In tonsillitis, croup, and other limited inflammations, if aconite is given in the earliest stage, when the chill is still on the patient, the skin, dry, and hot, and burning, soon becomes comfortably moist, and, in a short time, bathed in a profuse perspiration; and the quickened pulse becomes less frequent, and, from twenty-four to forty-eight hours, both the circulation and temperature become natural. A moderate dose, say 1 gtt. will make the pulse not only slower, but fuller and stronger, and more compressible. Hence its good effect in typhoidal fevers, characterized by depressed circulation—a want of freedom of the circulation. It may be given in doses of from ½ to 1 gtt. every hour, at first, and the intervals prolonged as it affects the heart's action.

